# Predictors of Mortality among Adult People Living with HIV and Its Implications for Appointment Spacing Model Approach Care

**DOI:** 10.4314/ejhs.v31i5.3

**Published:** 2021-09

**Authors:** Aderajew Nigusse Tekelehaimanot, Tefera Belachew, Esayas Kebede Gudina, Masrie Getnet, Demuma Amdisa, Lelisa Sena Dadi

**Affiliations:** 1 Department of population and Family health, Faculty of Public Health, Jumma University, Jimma, Ethiopia; 2 Departments of Internal Medicine, Jimma Medical Center, Jimma University, Jimma, Ethiopia; 3 Department of Epidemiology, Faculty of Public Health, Jumma University, Jimma, Ethiopia; 4 Department of Health Behavior and Society, Faculty of Public Health, Jimma University, Jimma, Ethiopia

**Keywords:** Adult, Appointment Spacing Modals, ART, PLWHA, Predictors, Jimma zone

## Abstract

**Background:**

Ethiopia has been motivated to achieve a goal of “appointment spacing model approach care.” However, little has been documented on the predictor of mortality and challenges of sustainable HIV care. Therefore, the aim of this study was to determine predictors of mortality among adult people living with HIV/AIDS on antiretroviral therapy (ART).

**Methods:**

A retrospective cohort study was conducted on 676 adult people living with HIV who enrolled to ART clinic from September 01, 2012 - August 30, 2016. Multivariable Cox Regression analysis was done where adjusted hazard ratio (AHR)with corresponding 95% confidence interval (CI) at P value ≤ 0.05 cut of point was used to identify predictors of mortality.

**Results:**

The total person-time contributed was 28,209 person-months with an overall mortality incidence rate of 11 per 1000 person-months observation. The cumulative mortality incidence among females over the study period was 16.8% (64/382). Severe undernourishment and moderate malnutrition at baseline, younger age, female sex, single, divorced, illiterate, lack of disclosure, advanced WHO clinical stage, seeking treatment outside catchment area, rural residence and immunological failure were found to be independent predictors of mortality.

**Conclusions:**

Poor nutritional status at baseline, advanced stage of HIV disease, occurrence of treatment failure, female sex, substance abuse, lack of social support, immunological failure, clinical failure, and younger age, low level of education and poor physical access to healthcare facility were found to be important predictors of mortality. Intervening, those factors as routine and part of the appointment spacing model care can improve survival of PLWHA.

## Introduction

Globally, between 1980 and 2015, about 78 million people were infected and half of them have died of AIDS (1, 2). According to the 2016 report of Global Burden of Diseases (GBD), worldwide 38.8 million people had HIV infection; 2.1 million people had been newly infected and 1.2 million died due to the virus in 2015 (3,4).

Ethiopia is among African countries hard hit by the HIV pandemic. The overall prevalence of the disease among adults, pregnant women and, women who had multi sexual partners was reported to be 0.9%, 0.4%, and more than 6%, respectively (5,6). The prevalence among women (1.2%) was twice that of men (0.6%), seven times in urban residence (2.9%) compared to rural settings (0.4%). In 2016, a total of 19,743 people died of AIDS and about 792,840 children lost either or both of their parents due to AIDS (7).

To alleviate the impact of HIV/AIDS, Ethiopia introduced ART in 2003 for the first time at cost of patients, and two years later ART started to be provided for free (8, 10, and 11). Following the 3 by 5 program, 535,069PLWHA who had ‘ever started’ ART and 375,811 PLWHA who did not yet started ART were put on ART in more than 1000 health facilities in 2014/5 (9). Subsequently, report of 2017 revealed that HIV incidence, AIDS related mortality and overall HIV prevalence declined by 95%, 73% and 29%, respectively (12).

Ethiopia has also been implementing prevention, treatment and care interventions. The prevention activities include voluntary counseling and testing (VCT), prevention of mother to child transmission (PMTCT), behavioral change communication and community mobilization. The treatment and care theme encompasses ART, laboratory monitoring, provider initiated testing and counseling (PITC), nutritional support, palliative care and home-based care activities (13).

Despite the efforts devoted to limit the impact of HIV, still numerous gaps and challenges remain unsolved. Among others, timely initiation of ART, retention in care and prevention of HIV related mortality, narrowing significant inequities in ART coverage, which varies between children (23%) versus adults (60%), females (54%) versus male (69%), and disparities among regions ranging from 5.6% to 93% are worth mentioning (14,15).

To help HIV patients fully benefit from ART, attain national goals and use resources most effectively, Ethiopia uses the HIV continuum care (HCC) framework initially created by the Centers for Disease Control and prevention (CDC) in Atlanta, USA, in 2013 (16). The HIV continuum of care is a series of stages from the time a person is diagnosed with HIV through assessment for ART eligibility, retention in care, and immunologic success and virological suppression via treatment adherence towards reduction of mortality and ART care success outcomes (17–19).

Many activities have been attempted to address negative ART care outcomes, nevertheless, there have been challenges at every stage of the HCC. These include late HIV care presentation (LP) (20), discontinuation of ART (21), poor ART adherence (22), immunologic (23), clinical (24), treatment (25) and virologyical failures (26).

Currently, Ethiopia is implementing a new care approach known as “appointment spacing model (ASM) care, which mainly focuses on a few visits of hospitals (two times per year) and comprehensive care at a time avoiding frequent laboratory and clinical monitoring. Hence, the approach enhances adherence and retention of ART cohorts in the treatment protocol, which in turn improves the maturity of immunity and virally suppresses viral load. Thus, the approach is thought to improve service quality, save costs, improve health outcomes, and accelerate the achievement of the 90-90-90 target by offloading workload from the already overburdened health facilities (4). However, there are still massive challenges pertaining to ART program regardless of the efforts being devoted by both governmental and nongovernmental organizations (27). Consequently, patients usually experience different unintended health care outcomes such as loss of many patients from the ART schedules (28), failure to recover their immunity (29) and deaths from the disease (30) to list a few.

The overall picture shows that research is lacking to identify key factors that lead to negative ART care outcomes, including mortality. Furthermore, studies conducted in Africa have revealed that factors affecting ART care are beyond individual level. The factors are related to health care institutions such as lack of ART trained health professionals (31), and lack of quality care (32); community level factors such as stigma (33) and traditional healer (34); and program level factors such as political commitment (35,36) and lack of coordinated HIV care activities (37–40). Nevertheless, comprehensive study has not been carried out in Ethiopia to adequately address the challenges of ART care and mortality from the viewpoints of stakeholders. Therefore, the aim of this study was to assess predictors of mortality among adult PLWHA in Jimma Zone Public Hospitals.

## Methods

**Study setting and period**: The study was conducted in the Jimma zone five public hospitals (Jimma Medical Center, Shenen Gibe, Agaro, Seka and Limmu Genet hospitals). Jimma zone is one of the 20 administrative zones in Oromia Regional State located in southwest Ethiopia. Jimma Medical Center is the only tertiary hospital in southwest Ethiopia. The other four hospitals are primary hospitals with catchment population of about a million each. At the time of the study, a total of 11,186 adults and 2,683 children were in chronic HIV care at the hospitals. The data was collected from February 15/2018 to March 16/2018 and the study involved patients on chronic HIV care at the hospitals during period of 01 September 2012 to 30 August 2016.

**Enrolment procedures/Retrospective cohort study:** A total of 676 adult PLWHA was randomly selected from adults (≥ 18 years old) who were on chronic care follow up. The Sample size was estimated using two population proportions formula. Based on BMI measurement, those PLWHA, who had undernourishments at the initiation of ART were considered to be exposed (n^1^) whereas those who had normal BMI were taken to be non-exposed group (n^2^). Accordingly, 281 exposed and 395 non-exposed samples were estimated, implying total of 676 samples of PLWHA for the study. Patients with unknown nutritional status at ART initiation, those who defaulted, those with incomplete information and those transferred out to other facilities were excluded from the study.

**Data source and collection procedure:** Medical records were reviewed from February 15/2018-murch 16/2018 covering a period of five years (from 01 September 2012 to 30 August 2016) using appropriate case reporting format prepared in English. Ten BSc professional nurses using data collection format collected the data. All the five hospitals have an electronic patient database called Comprehensive Care Centre Patient Application Database (C-PAD). C-PAD is an Electronic Medical Record (EMR) system database that contains patients' both clinical and non-clinical information. This was the main source of data in this study. Data were extracted using a data extraction checklist from the database. In case, data were incomplete, we tried to refer the patients' cards, registration and log books using patient medical record number and ART registration number.

**Data processing and analysis:** Cleaned and coded data were entered to EPI-data version 3.1.4, and then exported to SPSS version 20.0 for analysis. Kaplan Meier (KM) survival function and Log rank test was used to test the statistical difference in the KM curves. Bivariate Cox regression analysis was done to estimate the unadjusted Hazard Ratios (HRs). Independent variables with P ≤ 0.25 at bivariate analysis level were entered into the multivariable Cox regression model to control potential confounders. Finally, variables, which had P confidence intervals (CIs) at multivariable analysis, were considered independent predictors of mortality among the PLWHA.

**Ethical approval:** Waiver of the consent was obtained from the office of institutional ethical review board (IRB) of the Institute of Health Jimma University, and the reference number was (IHRPG/807/17). The data access permission was obtained from the Jimma zone five public hospitals board. We could not use any individual-level data from inception to this manuscript. The results were also maintained using aggregated report rather than individual characteristics in any aspect of the data throughout all steps and manuscript preparation. These data were not identifiable; therefore, informed consent was not required.

## Results

**Socio demographic characteristic of adult PLWHA in Jimma zone public hospitals**: In this study, records of 382 (56.5%) females and 294 (43.4%) males were reviewed. The mean ≤ 0.05 and non-null values within respective age of the study participants at the time of ART initiation was 30.4 years (± SD 7.4); 487 (72%) of them were between 25 and 44 years of age. More than half (55.6%) of them were married and 132 (19.5%) of them were divorced ones. Most of the participants (79.3%) reported to have attended at least a primary school. About 61% of the participants (412) were urban residents and 128 (18.9%) were living outside the catchment area of the respective hospitals. Most of them chew (80%) Kchat and more than half (58.6%) of the study participants reported to have used alcohol (Table 1).

**Table 1:** Socio-demographic characteristic of adult PLWHA in public hospitals, September 2012 – August 2016

Variables	Classification	Number	Percent
Sex	Male	294	43.4
	Female	382	56.5
Age group	18–24	97	14.3
	25–34	244	36.1
	35–44	243	35.9
	45–54	72	10.6
	>55	20	2.9
Marital status	Single	92	13.6
	Married	376	55.6
	Divorced	132	19.5
	Widowed	77	11.3
Level of Education	Illiterate	140	20.7
	Primary	271	40.1
	Secondary	190	28.1
	Collage	75	11.1
Residence	Urban	412	60.9
	Rural	264	39.1
Catchment area	within catchment	548	81.1
	out of catchment	128	18.9
Have Care giver	Yes	448	66.3
	No	228	33.7
Disclosure	Yes	467	68.9
	No	211	31.1
History using alcohol	Yes	397	58.6
	No	281	41.4
History of *kchat use*			
	Yes	540	79.6
	No	138	20.4

**Figure 1:**
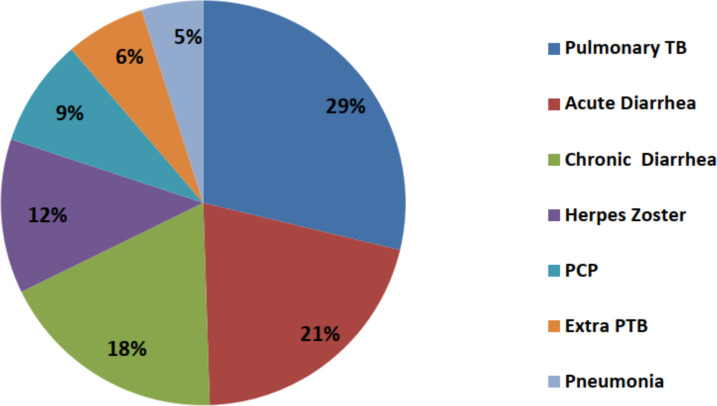
Opportunistic infection among adult PLWHA on ART, Jimma Zone public hospitals, September 2012 – August 2016.

Four hundred eighty-seven (72.0%) of the participants had history of opportunistic infections (OIs) at ART initiation. TB was the most common OI identified contributing for more than a third (35%) of the OIs cases (pulmonary TB – 29% and extra pulmonary TB – 6%). However, 71.6% of patients who were started on TB treatment were negative for Acid Fast Bacilli (AFB). The other OIs were diarrhea 101 (21.0%), chronic diarrhea (18.0%) and Herpes zoster (12.0%) (Figure 1)

**Cumulative Incidence and Incidence rate of mortality:** Ninety-eight (14.5%) patients died during the five-year of ART follow up period. The cumulative incidence among females was 16.8% (64/382). The total follow up time was 28,209 person-months when an overall incidence rate of 11 deaths per 1000 person-months was observed. The death rates among those PLWHAs who had severe and moderate malnutrition were 5.7 and 2.9 deaths per 1000 person-months, respectively.

The overall survival probability of the adult PLWHA was 86.6%. The overall estimated mean (±SD) survival time of adult PLWHA on ART follow up was 42.8 (SD = 17.8) months. In the five-year time interval, the highest mortality was observed during 18 to 24 months of follow up, followed by the months of 30 to 36 and 24 to 30, respectively. The lowest mortality was observed during the follow up period from 48 to 54 months followed by 54–60 months of follow up, followed and during the period 6 to 12 months (Table 2).

**Table 2:** Overall life table of PLWHA on ART follow up in Jimma Zone public hospitals southwest Ethiopia, September 2012 – August 2016

Interval	Beginning	Death/98	Lost/134	Survival	Sta/ error	95% CI	
0–6	676	10	0	0.9783	0.0042	0.9766	0.9941
6–12	666	5	0	0.8781	0.0056	0.9639	0.9867
12–18	661	8	34	0.9325	0.0091	0.9209	0.9570
18–24	608	19	13	0.9211	0.0106	0.8965	0.9386
24–30	558	14	12	0.9022	0.0118	0.8763	0.9230
30–36	561	17	23	0.8853	0.0129	0.8582	0.9091
36–42	521	9	25	0.8748	0.0137	0.8451	0.8992
42–48	487	11	16	0.8608	0.0158	0.7289	0.8874
48–54	476	1	10	0.8500	0.0159	0.8157	0.8782
54–60	470	4	2	0.8453	0.0565	0.7096	0.9748

**Figure 2:**
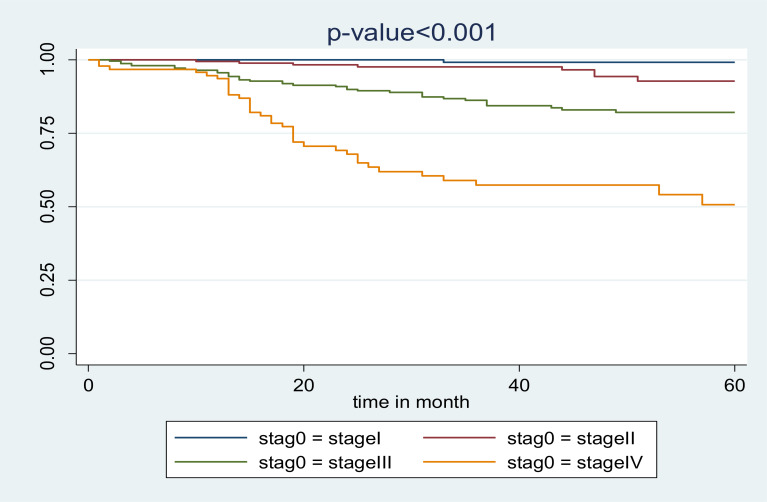
Kaplan Meier survival curve of PLWHA on ART follows up Jimma zone public hospitals by baseline WHO stages September 2012 – August 2016.

The overall probability of survival among adult PLWHA on follow up in Jimma zone public hospitals showed a decrement with an increment of WHO stages. Those PLWHA on ART follow up classified as WOH stage I, II, III and IV had a survival probability of 96.3%, 94.9%, 76.5% and 48.6%, respectively (Figure 2).

**PREDICTORS OF MORTALITY AMONG ADULT PLWHA ON ART FOLLOW UP:** Adult PLWHA who suffered from severe malnutrition at baseline had nearly four times (AHR: 3.7; 95% CI: 1.6, 6.7) risk of death while those with moderate malnutrition had more than twice (AHR: 2.5; 95% CI: 1.7, 7.5) risk of dying early compared to those with normal BMI. The risk of early death was nearly three times higher among females (AHR: 2.8; 95% CI: 2.1, 4.6) compared to males. Deaths among single (AHR: 2. 6; 95% CI: 1.8, 3.8), divorced (AHR: 2.4; 95% CI: 1.3, 3.9), and widowed (AHR: 2.3; 95% CI: 1.7, 3.7) PLWHA on ART follow up were higher compared to married PLWHA on ART follow up.

The risk of death among adults PLWHA decreases as educational status increases. The risk of death was more than two times among illiterate (AHR: 2.5; 95% CI: 1.9, 4.8) and those who attend primary school (AHR: 2. 1; 95% CI: 1.8, 3.6) compared to PLWHA on ART follow up who attend secondary or above schools.

Those PLWHA who did not disclose their HIV status to anyone (family or friend) had more than three times (AHR: 3.6, 95% CI: 1.7, 9.5) risk of dying compared to those who have disclosed their HIV status. Those adult PLWHA with TB Co-infection at the initiation of ART were nearly three times (AHR: 2.9,95% CI: 1.5, 5.5) more likely to die within five years of treatment follow up period compared to those who did not have it. Individuals who were at stage IV HIV diseases (bedridden) at the initiation of ART were more than three times (AHR:3.7,95% CI: 2.4,13.8) more likely to die within five years of treatment follow up period as compared to those with working functional status.

The risk of death was four times in those with elevated liver enzymes (>50 IU/L) (AHR: 4.2; 95% CI: 2.4, 6.7) and nearly three times higher in those with OI (AHR: 2.5; 95% CI: 1.9, 7.1) compared to their counterparts. Those PLWHA on ART follow up whose causes of death confirmed to be clinical failure (AHR: 2.1; 95% CI: 1.4, 2.8) and immunological failure (AHR: 1.7; 95% CI: 1.3, 2.7) were two times at higher risk of wasting syndrome (severe malnutrition /stage three malnutrition and leading to death) within five year treatment follow up period compared to their counterparts. The risk of death was also found to be high among PLWHA living outside catchment area of the hospital (AHR: 3.6; 95% CI: 1.5, 5.4) compared to their counterparts (Table 3).

**Table 3:** Predictors of mortality among Adult PLWHA in Jimma Zone public hospitals, Southwest Ethiopia, 2012 – August 2016

Variables	Classification	Death (%)	Censored (%)	CHR (95%)	AHR (95%)	P value
Age group	18–24	40(40.8)	57(9.8)	4.1 (1.5,11.3)	2.1 (1.7, 3.3)	0.01
	25–34	23(23.5)	221(38.2)	2.3 (1.8, 6.6)	0.8(0.3, 2.4)	0.04
	35–44	10(10.2)	232(40.1)	1	1	
	45–54	8(8.2)	64(11.1)	2.1 (1.4,16.3)	0.9 (0.4, 9.3)	0.38
	>55	17(17.3)	4(0.7)	3.7 (0.8, 6.6)	1.8 (0.8, 7.6)	0.02
Sex	Male	34(34.7)	260(44.9)	1	1	
	Female	64(65.3)	318(55.0)	3.1(1.5, 5.5)	2.8 (2.1,4.6)	0.03
Marital status	Married	9 (9.2)	36(6.2)	1	1	
	Single	56(57.1)	367(63.5)	4.3(2.0, 7.9)	2.6 (1.8, 3.8)	0.01
	Divorced	14 (14.3)	118(20.4)	4.1(2.2, 7.2)	2.4 (1.3, 3.9)	0.02
	Widowed	19 (19.4)	58(10.0)	3.6 (2.5, 6.7)	2.3 (1.7, 3.7)	0.03
Education Level	Illiterate	53(54.1)	87(15.1)	5.7(3.3,9.8)	2.5 (1.9, 4.8)	0.00
	Primary(1–8)	26 (26.5)	242(41.9)	2.5(1.6, 6.7)	2.1 (1.8, 3.6)	0.01
	Secondary(9–12)	15 (15.3)	24(4.2)	1.5 (1.1, 5.3)	0.5 (0.3, 3.6)	0.21
	college	4(4.1)	71(12.3)	1	1	
BMI at initiation of ART	Normal	6 (6.1)	240(41.5)	1	1	
	Moderate malnutrition	31(3.1)	298(51.6)	3.6 (2.3,9.4)	2.5(1.7, 7.5)	0.01
	Severe malnutrition	61(6.1)	40(6.9)	5.6 (4.3,8.3)	3.7 (1.6, 6.7)	0.01
Functional status	Working	29 (29.6)	372(64.4)	1	1	
	Ambulatory	37 (37.8)	189(32.7)	1.3 (1.9, 3.6)	1.04(0.9, 2.2)	0.04
	Bedridden	32 (32.6)	17 (2.9)	6.1 (4.7,18.6)	3.7(2.4,13.8)	0.03
TB-co infection	No TB	19 (19.4)	224(38.8)	1	1	
	INH Prophylaxis	21 (21.4)	172 (29.8)	1.2 (0.8, 3.1)	1.1(0.7, 2.9)	0.04
	TB treatment	58 (59.2)	182(31.5)	4.5 (3.5, 7.8)	2.9(1.5, 5.5)	0.02
WHO clinical stage	Stage I&II	8(8.2)	394(68.2)	1	1	
	Stage III	58 (59.2)	177(30.6)	3.5(2.1, 5.4)	1.7 (1.4, 3.2)	0.01
	Stage IV	32 (32.6)	7 (1.2)	7.1(3.8, 11.8)	3.7 (1.7, 5.3)	0.00
CD4 count	<200	57 (58.2)	301 (52.1)	4.2 (1.5,10.3)	2.3(1.8, 6.3)	0.01
	201–350	28 (28.6)	221 (38.2)	2.3 (0.8, 6.6)	1.7(0.6, 4.9)	0.02
	>350	13 (13.2)	56 (9.7)	1.60(1.04, 2.)	1	
follow up within catchment	yes	19 (19.4)	524(90.7)	1	1	
	No	79(80.6)	54(9.3)	6.9 (3.9, 9.2)	3.6 (1.5, 5.4)	0.01
Residence	Urban	24 (24.4)	338(58.5)	4.10(1.5,11.3)	1	
	Rural	74 (75.6)	240(41.5)	2.3 (0.8, 6.6)	2.1(1.4, 3.3)	0.02
Alcohol and Kchat use	Yes	68(69.4)	329(56.9)	3.6 (1.1,2.6)	1.8 (1.4, 5.4)	0.03
	No	30(30.6)	249(43.1)	1	1	
Patient Status	Not Known	12 (12.2)	156(26.9)	1	1	
	Clinical failure	36 (36.7)	142(24.6)	4.1 (1.5,10.3)	2.1 (1.4, 2.8)	0.02
	Immunologic failure	24 (24.5)	127(21.9)	2.4(0.7, 5.6)	1.7 (1.3, 2.7)	0.02
	Virologic Failure	26 (26.5)	153(26.5)	2.8(1.3,3.7)	1.7 (1.3, 2.3)	0.02
Liver Function test	Normal (0–50)	29(29.5)	299(51.2)	1	1	
	Abnormal (>50)	69(70.4)	284(49.1)	6.4(3.8, 7.4)	4.1(2.4, 6.7)	0.01

N.B: HR, hazard ratio; CI, confidence interval; ART, antiretroviral therapy; WHO, World Health Organization; BMI, body mass index

## Discussion

ART Program has created a significant change in improving both health status and life expectance of PLWHA. However, first-line drug resistance and death from HIV related causes are quiet formidable challenges in Sub-Saharan Africa, including Ethiopia. In this study, 14.9% cumulative incidence of HIV mortality was reported in patient on ART. This is lower than finding of a study conducted in Cameroon: (18). The variation could be due to the fact that in the earlier study, participants were recruited both from health centers and hospitals where the service from health Center was not comprehensive as Hospital.

The study revealed that mortality was found to be higher among adult PLWHA (65.3 %). This finding is in line with a study conducted Northwest Ethiopia, in which the prevalence of HIV infection among females was two times higher than males (19). Studies from African countries also support this finding. The prevalence of HIV related death among female and male was reported to be 6.5% and 4.7% in Sub-Saharan Africa (28), and 7.5% and 4.3% in china (29), respectively. The fact that rates of HIV infection and death among women are higher imply not only gender difference but also gender inequality, leaving women to more vulnerable impact of HIV/AIDS, which calls for the need for strengthening HIV care and support more among females to curb the challenge.

In this study, the highest number of deaths was noticed in the 2^nd^ and 3^rd^ years of follow up. This could be attributed to eligibility criteria, change of guideline for ART initiation and project driven approach care as well as using test and treat immediately, regardless of CD4 count and WHO clinical staging (30,31). The other reason may be Ethiopia doesn't implement strict follow up care (33) for patients with good adherence at 1^st^ and 2^nd^ year that trust in and awareness of modern medicine was poor, and conversely high in traditional medicine, which patients could consider as an alternative option (34, 35).

The presents of nutrition by prescription program and accessibility of ART was deprived during the period as compared to today. On the other hand, mortality declined in years 2015 and 2016 as compared with earlier years. This might be because of improvement, accessibility and awareness of ART users as evidence by findings of studies done in Northwest Ethiopia and Asian countries/Nepal (36, 37).

Marital status was significantly associated with the survival status of ART users. Those who were not married were more affected by HIV related death compared to married ones. This finding is similar to finding of a studies done in developing countries (24, 28, 38). This might be due to the fact that those adult PLWHA who were out of marriage were at higher risk of exposure to unhealthy lifestyles, as well as they might have been challenged with stigma and discrimination emanating from the community, which can in turn lead to poor compliance to treatment.

Lack of or minimal participation in any working opportunity to generate income can push them to more risks compared to individuals who have social and family support. On the other hand, those who were not married might have wider sexual network, which leads to super infection of HIV and repeated infections of sexually transmitted diseases that can result in double burden increasing risk of mortality.

In this study, mortality was higher among adult PLWHA who had developed immunologic failure, WHO clinical stages III and IV, low CD4 count, severe and moderate malnutrition, poor functional status and abnormal liver function test results compared to their counterparts in the five year follow up period. These findings are in line with other findings from middle and low-income countries (11, 26, 19, 39, 40).

This indicates that those vulnerable PLWHA to having an advanced stage of the disease are usually accompanied by multiple challenges, co-morbidities and humble prediction of outcome. In addition, such outcome indicates a very low performance to meet the expected UNAIDS targets for Ethiopia by 2020 in spite of the fact that Ethiopia has launched “appointment spacing model approach care (few/two visit per year of clinics by PLWHA)”.

In conclusion, the incidence of HIV mortality was 11 deaths per 1000 person-months, whereas most deaths (20%) occurred during the 18–24 month follow up period interval. Females are at higher probability of dying from AIDS related causes. Marital status, functional status, education level, disclosure of HIV status, having care givers/support, abnormal test of liver function, presence of opportunistic infection, malnutrition were found to be independent predictors of AIDS related mortality.

These findings imply that applying an action/intervention towards these leading cases of mortality as routine and as part of the new approach care “appointment spacing model care (ASMC)” can reduce mortality and can be a sustainable clinical care other than project driven intervention of HIV care. Further longitudinal study that explores on how to address real livelihood and methods of tracing are recommended.

Finally, it is price mentioning some weaknesses of this study. First, mortality might be underestimated, since patients lost to follow-up probably include individuals dying at home without being reported. Additionally, since secondary data was used for this study, it was impossible to include some key variables such as economic status and psychological distress that need to be included in this study.

There was also incompleteness of records for some of the patients enrolled on ART. On the other hand, because the study was done during the time when Ethiopia is striving to achieve its recently planned “with the new approach of ASMA,” the findings may give better insights into the problems that shall be considered to achieve the goal.
